# Validation of Clinical-Grade Electroporation Systems for CRISPR-Cas9-Mediated Gene Therapy in Primary Hepatocytes for the Correction of Inherited Metabolic Liver Disease

**DOI:** 10.3390/cells14100711

**Published:** 2025-05-14

**Authors:** Justin Gibson, Abishek Dhungana, Menam Pokhrel, Benjamin Arthur, Pramita Suresh, Olumide Adebayo, Renee N. Cottle

**Affiliations:** Department of Bioengineering, Clemson University, Clemson, SC 29634, USA; jrg6@g.clemson.edu (J.G.); adhunga@clemson.edu (A.D.); mpokhre@clemson.edu (M.P.); bbarthu@clemson.edu (B.A.); psuresh@g.clemson.edu (P.S.); oadebay@g.clemson.edu (O.A.)

**Keywords:** CRISPR-Cas9, clinical translation, gene therapy, therapeutic liver repopulation, electroporation

## Abstract

Hepatocyte transplantation (HTx) combined with ex vivo gene therapy has garnered significant interest due to its potential for treating many inherited metabolic liver diseases. The biggest obstacle for HTx is achieving sufficient engraftment levels to rescue diseased phenotypes, which becomes more challenging when combined with ex vivo gene editing techniques. However, recent technological advancements have improved electroporation delivery efficiency, cell viability, and scalability for cell therapy. We recently demonstrated the impacts of electroporation for cell-based gene therapy in a mouse model of hereditary tyrosinemia type 1 (HT1). Here, we explore the use of the clinical-grade electroporator, the MaxCyte ExPERT GTx, utilized in the first FDA-approved CRISPR therapy, Casgevy, and evaluate its potential in primary hepatocytes in terms of delivery efficiency and cell viability. We assessed the gene editing efficiency and post-transplantation engraftment of hepatocytes from *mTmG* mice electroporated with CRISPR-Cas9-ribonucleoproteins (RNPs) targeting 4-hydroxyphenylpyruvate dioxygenase (*Hpd*) in a fumarylacetoacetate hydrolase (*Fah*)-deficient mouse model of HT1. After surgery, *Fah^-/-^* graft recipients were cycled off and on nitisinone to achieve independence from drug-induced Hpd inhibition, an indicator of HT1 disease correction. Transplanted hepatocytes subjected to electroporation using the GTx system had a cell viability of 89.9% and 100% on-target gene editing efficiency. Recipients transplanted with GTx-electroporated cells showed a smaller weight reduction than controls transplanted with untransfected cells (7.9% and 13.8%, respectively). Further, there were no mortalities in the GTx-recipient mice, whereas there was 25% mortality in the control recipients. Mean donor cell engraftment was significantly higher in GTx-recipient mice compared to untransfected control recipients (97.9% and 81.6%, respectively). Our results indicate that the GTx system does not negatively impact hepatocyte functionality and engraftment potential, thereby demonstrating the promise of GTx electroporation in hepatocytes as a viable cell therapy for treating genetic diseases that affect the liver.

## 1. Introduction

Inherited metabolic liver diseases (IMLDs) are characterized as genetic diseases caused by the loss of function in liver-specific enzymes or transport proteins, resulting in impaired metabolic pathways [1]. Although orthotopic liver transplantation (OLT) is the gold standard treatment for advanced stages of uninherited chronic and acute liver diseases as well as IMLDs, shortages of quality donor organs, high costs of treatment, and risks of organ rejection are pushing the scientific community towards alternate therapeutic approaches [2,3].

With advancements in gene therapy, the combination of hepatocyte transplantation (HTx) with ex vivo gene therapy represents a promising cell-based therapeutic approach for treating IMLDs [4,5]. Combining HTx with gene therapy has the potential to enable selective expansion of transplanted hepatocytes in vivo, thereby restoring hepatic function [6,7]. For the past 40 years, HTx has been widely studied to treat liver-based disorders [8,9,10,11]. Compared to OLT, HTx is a less invasive procedure requiring fewer donor resources for effective treatment and could potentially utilize hepatocytes isolated from the patient’s resected liver, eliminating the need for lifelong immunosuppression therapy.

The capacity of CRISPR-Cas9 to induce precise double-strand breaks at target genomic loci is the fundamental basis for gene editing [12]. The development of CRISPR technology has ushered significant possibilities for the advancement of gene therapy. In that regard, viral-vector-based CRISPR-Cas9 therapies, such as those that use adeno-associated virus (AAV) vectors, have been widely employed for the treatment of IMLDs, including phenylketonuria, Wilson’s disease, and familial hypercholesterolemia [13,14,15]. However, pre-existing immunogenicity, limited packaging capacity, risk of insertional mutagenesis, and off-target organ transfection have significantly hindered AAVs as a candidate for CRISPR-Cas delivery [16,17]. Incidence of hepatocellular carcinoma (HCC), for example, has been reported as a side effect of in vivo liver-directed AAV gene therapy [18,19,20,21]. Furthermore, the ability of AAVs to remain as stable episomes in vivo has been shown to result in long-term Cas9 expression, eliciting off-target activity and potentially contributing to anti-Cas9 immunogenic reactions [22]. These limitations emphasize the importance of exploring non-viral delivery mechanisms for gene therapy.

Casgevy (exagamglogene autotemcel) is the first FDA-approved CRISPR-Cas9-based ex vivo gene therapy for sickle cell disease (SCD) and β-thalassemia. Casgevy involves electroporation to deliver CRISPR-Cas9 into hematopoietic stem and progenitor cells isolated from the patient’s peripheral blood, showing significant potential for long-term treatment [23]. The advantage of ex vivo gene editing therapies, such as Casgevy, is that there is no patient exposure to the CRISPR-Cas9 reagents. This allows for safer and more precise control over Cas9-mediated gene modification, eliminating the risk of transfection to off-target organs [24]. Although extensive processing steps consisting of patient cell isolation, gene editing, cryopreservation, and testing are required in ex vivo approaches, protocol optimization enables the effective use of such methods, ensuring high reproducibility and consistency [5].

In this work, we explore the use of a clinical-grade electroporation system, the MaxCyte ExPERT GTx, utilized in Casgevy therapy and evaluate its potential in primary hepatocytes for the delivery of 4-hydroxyphenylpyruvate-dioxygenase (*Hpd*)-aiming Cas9 ribonucleoproteins (RNPs), followed by HTx to rescue a mouse model of hereditary tyrosinemia type 1 (HT1). We demonstrate that the GTx system is a safe and effective ex vivo delivery system for gene editing in hepatocytes as a viable cell therapy approach for treating IMLDs.

## 2. Materials and Methods

### 2.1. Animal Care and Husbandry

All animal experiments were approved by and performed in accordance with the Institutional Animal Care and Use Committee regulations of Clemson University (Assurance number: D16-00435; approved on 24 June 2024). Donor hepatocytes were isolated from B6.129(Cg)-Gt (ROSA)26 Sor^tm4(ACTB-tdTomato, -EGFP) Luo^/J (*mTmG*) male mice, 6–8 weeks old, obtained from The Jackson Laboratory (JAX stock #007676). C57BL/6J *Fah*∆exon5 mice (*Fah^-/-^*), males 6 weeks old, were used as recipients of hepatocyte transplantation. The *mTmG* mice are genetically engineered to express a fluorescent protein, tdTomato, in cells and tissues, making tracking their engraftment in the liver easier. *mTmG* mice were housed no more than 3 to a cage. Recipient *(Fah^-/-^*) mice were fed a high-energy chow diet (5LJ5, PicoLab) and water containing 2-(2-nitro-4-trifluoromethyl benzoyl)-1,3-cyclohexanedione (NTBC or nitisinone) at a concentration of 8 mg/L (A384235, Ambeed, Arlington Hts, IL, USA). *Fah^-/-^* mice were housed no more than 2 to a cage following transplantation. *Fah^-/-^* mice taken off NTBC develop acute liver failure as toxic metabolites build up in the liver due to Fah deficiency. Treatment with NTBC rescues *Fah^-/-^* mice by inhibiting Hpd to block the accumulation of hepatotoxic metabolites upstream of Fah. The transplant recipient mice were monitored daily for changes in activity, weight loss, and overall health. A total of 18 mice were used as experimental units in this study.

### 2.2. Hepatocyte Isolation

The livers of *mTmG* donor mice were harvested under anesthesia. The right and left lateral lobes were perfused using the gentleMACS Liver Perfusion Kit (130-128-030, Miltenyi Biotec, Gaithersburg, MD, USA) followed by dissociation and hepatocyte isolation using a gentleMACS Dissociator in accordance with the manufacturer’s protocol. Following isolation, the hepatocytes were resuspended in 5 mL of cold HMX media, and the cell density and viability were quantified by trypan blue staining using a hemocytometer. Hepatocytes with a viability greater than 70% were used in the electroporation experiments. HMX media were prepared by combining DMEM high glucose with GlutaMAX (11995065, Thermo Fisher Scientific, Waltham, MA, USA), 10% fetal bovine serum (F2442, Sigma-Aldrich, Burlington, MA, USA), 100 U/mL penicillin, 0.1 mg/mL streptomycin (0.1 mg/mL streptomycin: 30002CI, Corning, NY, USA), and 10 MM HEPES (BP299-1, Fisher Scientific, Waltham, MA, USA).

### 2.3. Electroporation of CRISPR-Cas9 into Primary Hepatocytes

To determine the optimal electroporation program in the MaxCyte ExPERT GTx for cargo delivery in primary mouse hepatocytes, we tested electroporation programs with increasing voltage power output: THP-1, Optimization-4, Optimization-6, and Hepatocyte-3. Hepatocytes were isolated from the livers of two *mTmG* donor mice and pooled before treatment. For each program, three electroporation treatments were performed. The first treatment consisted of no cargo. The second treatment consisted of 10 μg CleanCap 5 moU eGFP mRNA (L-7201, TriLink Biotechnologies, San Diego, CA, USA). The third treatment consisted of *Hpd*-aiming CRISPR-Cas9 RNPs. Electroporation was performed using the GTx system with the following conditions per reaction: 1.2 × 10^6^ cells, 20 μL MaxCyte Electroporation Buffer (EPB-1); 0.3 μL *Hpd* sgRNA at a concentration of 20 μg/µL (IDT); 1.0 μL of 61 μM V3 SpCas9 (1081059, IDT, Coralville, IA, USA). The *Hpd*-sgRNA contained the same guide sequence used in Rathbone et al., 2022 [5].

A single pool of isolated hepatocytes was used for the transplant studies to reduce variability. Electroporation using the GTx system was performed using the following conditions for each reaction: 4.8 × 10^6^ cells, 100 μL EPB-1; 1.5 μL of 20 μg/μL Hpd sgRNA (IDT); and 4.9 μL of 61 μM V3 SpCas9 (1081059, IDT). To benchmark the GTx performance, freshly isolated hepatocytes were separately electroporated using a Lonza 4D Nucleofector system with program code CM-150. Electroporation on the 4D Nucleofector was performed with the following conditions per reaction: 1.2 × 10^6^ cells, 100 μL P3 buffer (V4XP-3024, Lonza, Bend, OR, USA); 1.5 μL of 20 μg/μL Hpd sgRNA; and 4.9 μL of 61 μM V3 SpCas9 (1081059, IDT). Promptly after electroporation, the hepatocytes were resuspended in cytokine recovery media to enhance their viability, as described in [25].

Hepatocytes were plated on six-well Corning BioCoat collagen I-coated plates (356400, Corning) at 300,000 cells per well. A 0.25 g/L collagen basement membrane matrix (356234, Corning) was overlaid 24 h following plating. The cells were imaged 24 h after plating using a Keyence fluorescence microscope (BZ-X810, Keyence, Itasca, IL, USA). The cell confluency and GFP fluorescence were quantified using the BZ-H4C software. GFP intensity values were calculated by dividing the measured GFP intensity by the measured confluency. Conditioned media were collected from the plated cells at 24 h after plating to quantify the secreted albumin using an AssayPro Mouse Albumin AssayMax ELISA kit (EMA3201-1, AssayPro, St. Charles, MO, USA). At 24 h after plating, the hepatocytes were assessed for cell viability using a CyQUANT MTT Cell Viability Assay (V13154, Thermo Fisher Scientific). DNA was isolated 3 days after plating to quantify the on-target Cas9 indel efficiency using the methods described in [25]. The sequencing methods employed blinding.

### 2.4. Hepatocyte Transplantation into Fah^-/-^ Mice

Recipient *Fah^-/-^* mice were switched to water without NTBC three days before transplantation. Recipient *Fah^-/-^* mice were randomly assigned to the following treatment groups: the 4D electroporation group (*n* = 4), the GTx electroporation group (*n* = 4), and the untransfected group (*n* = 4). For each *Fah^-/-^* transplant recipient, 500,000 viable electroporated hepatocytes were resuspended in 120 µL room temperature HMX medium. Within 2 h after electroporation, the cells were injected into the spleen of each recipient. Surgery was performed with mouse identification blinding. The untreated *Fah^-/-^* group (*n* = 3) served as no-surgery controls and were kept on NTBC. The transplanted mice were switched to NTBC water once a 10% weight loss was observed and maintained on NTBC water until their weights returned to baseline. After their weight recovered, recipient mice were switched to water without NTBC. The mice were cycled off and on NTBC until their weights remained stable independently of NTBC administration. Mice that did not survive the NTBC cycling were excluded from subsequent analyses, except for the survival analysis. We reasoned that including data for premature deaths would skew downstream assessments in successfully engrafted individuals.

### 2.5. Hepatocyte Engraftment Quantification

Recipient mice were anesthetized, and the livers were harvested from recipient mice after euthanasia. The right lateral lobes from transplant recipients and no-surgery controls were used to isolate hepatocytes, which were analyzed for tdTomato fluorescence using a Cytoflex S flow cytometer (Beckman Coulter, Brea, CA, USA). The flow cytometry gating strategy excluded debris and cell clumps. The gating strategy for hepatocytes with tdTomato fluorescence was determined using the untreated controls. The proportion of tdTomato-positive hepatocytes was used to quantify liver engraftment.

### 2.6. Histology and Immunohistochemistry Staining

The right and left medial liver lobes from the liver of euthanized mice were used for histology. From each lobe, ~3 MM thick sections were prepared and fixed in 10% neutral-buffered formalin (HT501640, Millipore Sigma, St. Louis, MO, USA). The sections were stained with hematoxylin and eosin (H&E) and anti-Fah antibody immunohistochemistry (IHC) staining, as described in [25]. Staining was performed in a blinded manner. The stained slides were imaged using a Leica DFC7000T camera on a Leica Laser Microdissection Microscope.

### 2.7. Metabolic Analysis

For the endpoint biomarker analysis, blood was collected by cardiac puncture. The serum was separated by centrifugation and analyzed for metabolic biochemical markers albumin (ALB), alkaline phosphatase (ALP), alanine aminotransaminase (ALT), total bilirubin (TBIL), creatinine (CREA), and glucose (GLU) using the 63,772 Custom Chemistry Panel (IDEXX BioAnalytics, North Grafton, MA, USA). This data collection was blinded.

### 2.8. Statistical Analysis

All statistical analyses were performed on GraphPad Prism 10.3.1. The Shapiro–Wilk test and Q-Q plot analysis were performed on each sample group to determine distribution normality. If groups tended towards a lognormal distribution, the Kruskal–Wallis one-way ANOVA with Dunn’s multiple comparisons was performed. If groups took a normal distribution, the Brown–Forsythe and Welch one-way ANOVA with Dunnett’s T3 multiple comparisons was performed. Statistical significance was denoted as * for *p* < 0.05, ** for *p* < 0.01, and *** for *p* < 0.001. The bar on all bar graphs displays the median, with the error bars displaying the range.

## 3. Results

### 3.1. GTx Electroporation Optimization

To determine the optimal electroporation program for the MaxCyte ExPERT GTx electroporator system, four pre-programmed voltage codes with gradually increasing power output were used. There were no notable differences between the four programs in cell confluency, viability, or functionality, as indicated by the MTT assay and albumin concentrations (Supplementary Figure S1A–C). Hepatocyte-3 provided the highest delivery efficiency, corresponding to 86.3% GFP-positive cells compared to 40% or less for the other programs (Supplementary Figure S1D,E). Each program provided 100% on-target indel editing efficiency (Supplementary Figure S1F). Because Hepatocyte-3 showed the highest delivery efficiency, we proceeded with this program for subsequent experiments.

### 3.2. Transplantation of Hepatocytes Electroporated with GTx into Fah^-/-^ Mice

**Figure 1:**
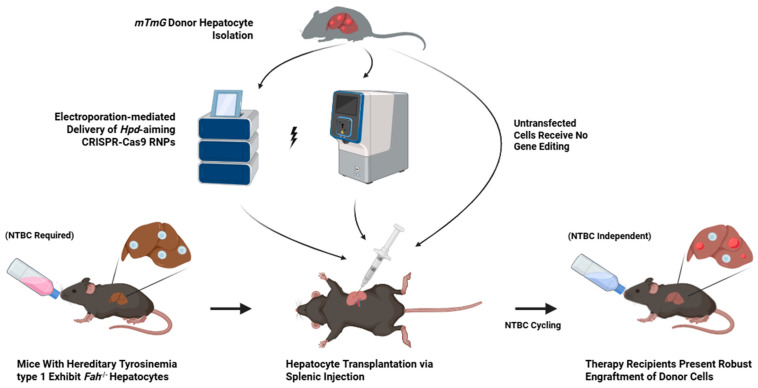
Schematic of therapeutic procedure. Healthy hepatocytes were isolated from mTmG donor mice and electroporated with Hpd-aiming CRISPR-Cas9 RNPs. Following electroporation, donor cells were transplanted via splenic injection into Fah^-/-^ mice. Recipient mice were cycled off and on nitisinone until independence from the drug was achieved. Abbreviations: Hpd, hydroxyphenylpyruvate dioxygenase; RNP, ribonucleoprotein; Fah, fumarylacetoacetate hydrolase.

**Figure 2:**
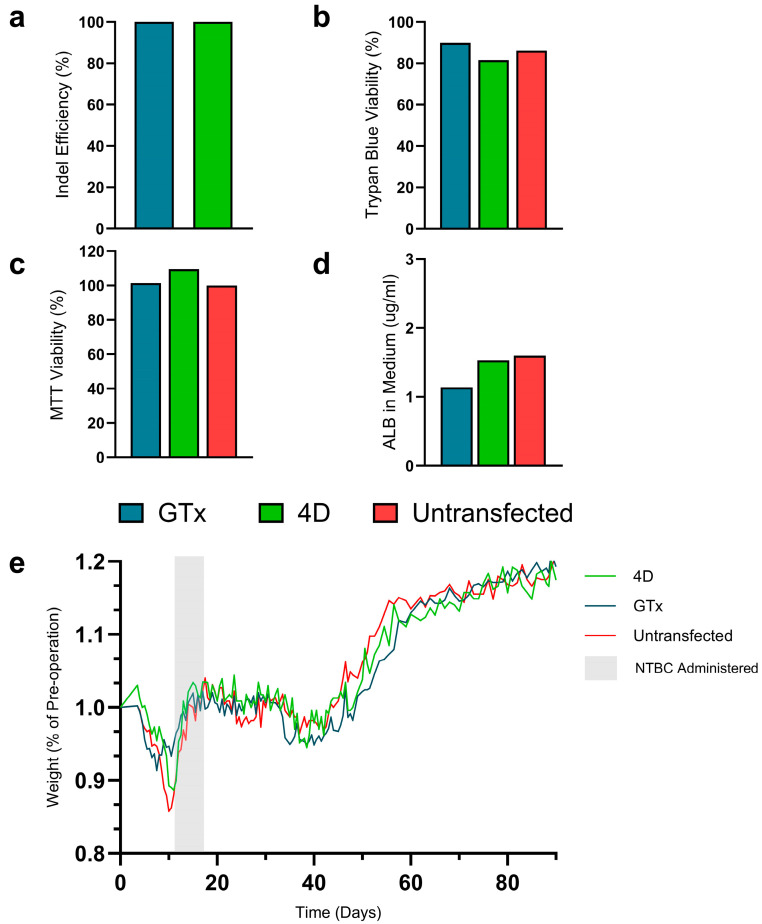
In vitro analyses of transplanted donor hepatocytes post-electroporation and recipient body weights post-transplantation. On-target gene editing efficiencies (**a**) measured in donor hepatocytes three days after electroporation (*n* = 1). Cell viability in hepatocytes (*n* = 1) was quantified by trypan-blue staining and cell counting and normalized to the viability of untreated hepatocytes (**b**). MTT assay viability measurements in plated hepatocytes after electroporation normalized to untreated controls (**c**) and albumin levels in conditioned media (**d**) at 24 h after plating. Bar graphs display individual values. Recipient body weights as a percentage of pre-operation weight over the entire selection period (**e**). For (**e**), the initial *n* = 4, there was one mortality in the untransfected recipients on day 9 and one in the 4D recipients on day 11. Abbreviations: ALB, albumin.

We previously demonstrated the electroporation-mediated delivery of CRISPR-Cas9 to achieve therapeutic liver repopulation to rescue the *Fah^-/-^* mouse model for hereditary tyrosinemia type 1 [25]. In this study, we isolated hepatocytes from *mTmG* donor mice and delivered *Hpd*-aiming Cas9 RNPs via electroporation into freshly isolated hepatocytes using the GTx (Figure 1). To benchmark the electroporation performance of the GTx system, we separately electroporated *mTmG* hepatocytes using the Lonza 4D Nucleofector system. Within two hours after electroporation, the hepatocytes were transplanted into *Fah^-/-^* mice via splenic injection to evaluate their engraftment potential. We transplanted untreated *mTmG* hepatocytes into the *Fah^-/-^* mice, which are referred to as untransfected controls. High on-target editing efficiency of 100% was observed for the GTx- and 4D-electroporated hepatocytes after plating (Figure 2a), and no notable differences in cell viability or functionality were observed between the electroporated cells and the untransfected controls (Figure 2b–d). The transplant recipient mice were taken off NTBC until a 10% reduction in body weight occurred 11 days after surgery to induce hepatotoxicity and activate the clonal expansion of grafted hepatocytes in the liver. At this point, NTBC was re-administered for the recipients to regain their baseline body weight and then NTBC was removed to continue liver repopulation by transplanted hepatocytes. Only one cycle of NTBC re-administration was required for all recipients before their body weights stabilized, independent of drug-induced Hpd inhibition (Figure 2e). All graft recipients treated with GTx-electroporated hepatocytes survived treatment, whereas there was one mortality in both the 4D-electroporated and untransfected recipient groups (Supplementary Figure S2). Further, the trend in body weight loss in recipients treated using the GTx-electroporated cells was less severe in their weakest state during the NTBC withdrawal period than the 4D and untransplanted recipient mice, indicating faster recovery from hepatotoxicity mice grafted with hepatocytes electroporated using the GTx system. Before re-administering NTBC, the untransfected recipient mice experienced a 13.8% loss in body weight, the 4D recipients showed a 9.8% decline, and the GTx recipients had a 7.9% reduction compared to baseline measurements (Figure 2e). The recipient mice were kept off NTBC for roughly 70 days to validate long-term recovery. During this time, all surviving recipients gained significant weight (Figure 2e), indicating sufficient liver repopulation to rescue mice from acute liver failure.

### 3.3. Therapeutic Outcomes in Recipient Mice After Liver Repopulation

**Figure 3:**
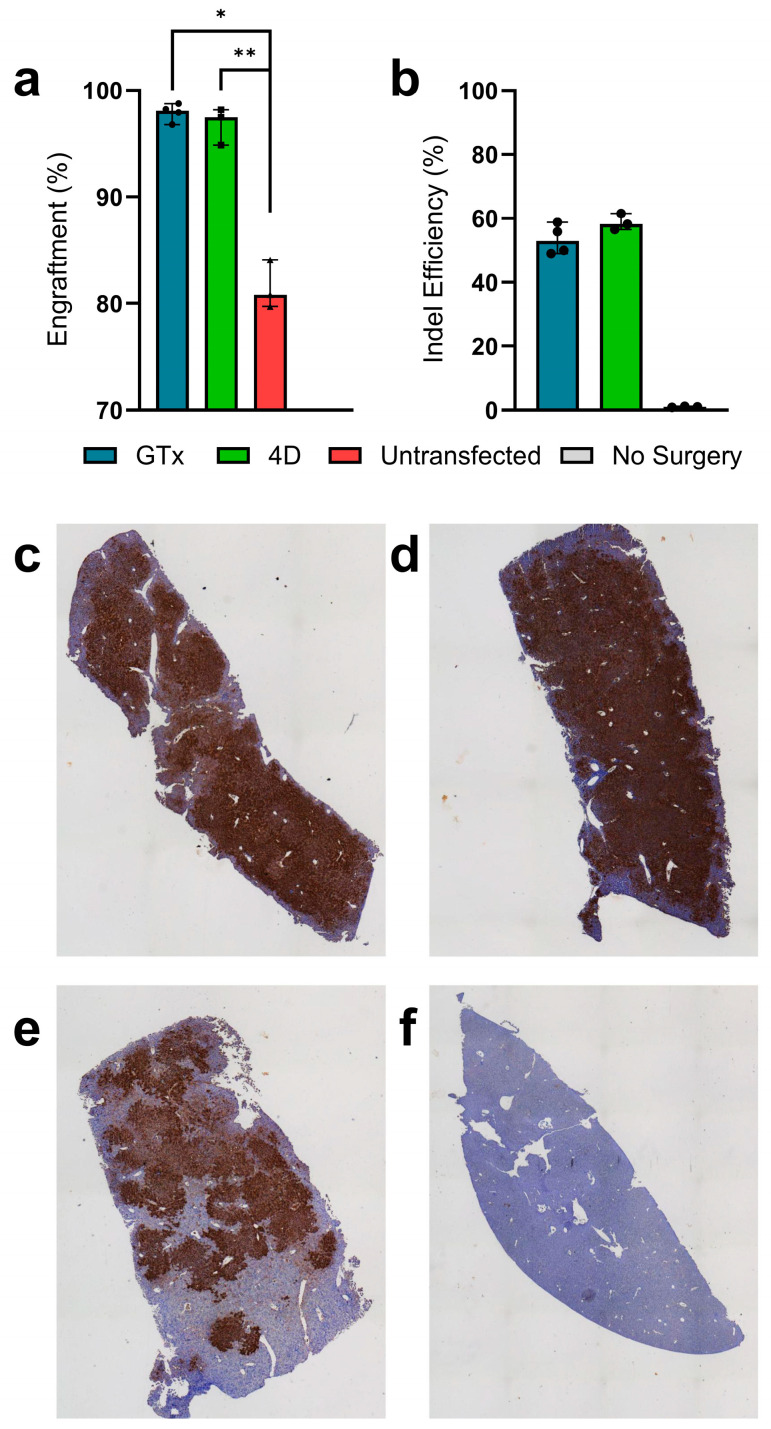
Liver repopulation and therapeutic efficacy in *Fah^-/-^* recipients. Measures of donor cell engraftment by tdTomato fluorescence of isolated hepatocytes using flow cytometry (**a**) and quantification of *Hpd* indel mutations of homogenized liver tissue (**b**) (*n* = 3–4 for recipients and *n* = 2 for untreated *Fah^-/-^* controls). Representative liver images subjected to anti-Fah IHC staining for recipients transplanted with (*n* = 4) GTx (**c**), (*n* = 3) 4D (**d**), and (*n* = 3) untransfected control (**e**) hepatocytes. IHC staining in untreated *Fah^-/-^* mice on NTBC (*n* = 2) was shown as a negative control to confirm proper staining (**f**). Bar graphs display the median and biological replicates, and error bars display the range. Statistical significance was denoted as * for *p* < 0.05 and ** for *p* < 0.01. Abbreviations: *Hpd*, hydroxyphenylpyruvate dioxygenase; *Fah*, fumarylacetoacetate hydrolase; IHC, immunohistochemistry; NTBC, 2-(2-nitro-4-trifluoromethylbenzoyl)-1,3-cyclohexanedione.

At the end of the 90-day selection period, the recipients were euthanized to quantify engraftment and the impacts of hepatocyte transplantation. The liver was dissected and divided for subsequent analyses. The gross liver anatomy appeared healthy, with no visible tumors in any recipients (Supplementary Figure S3). To assess engraftment, the right-lateral lobe of the liver was used to isolate hepatocytes, followed by flow cytometry to quantify tdTomato-positive hepatocytes. Both the GTx (97.9% and 81.6%, respectively, *p* = 0.0147) and 4D (96.8% and 81.6%, respectively, *p* = 0.0020)-treated mice showed significantly higher mean engraftment than the untransfected controls (Figure 3a).

**Figure 4:**
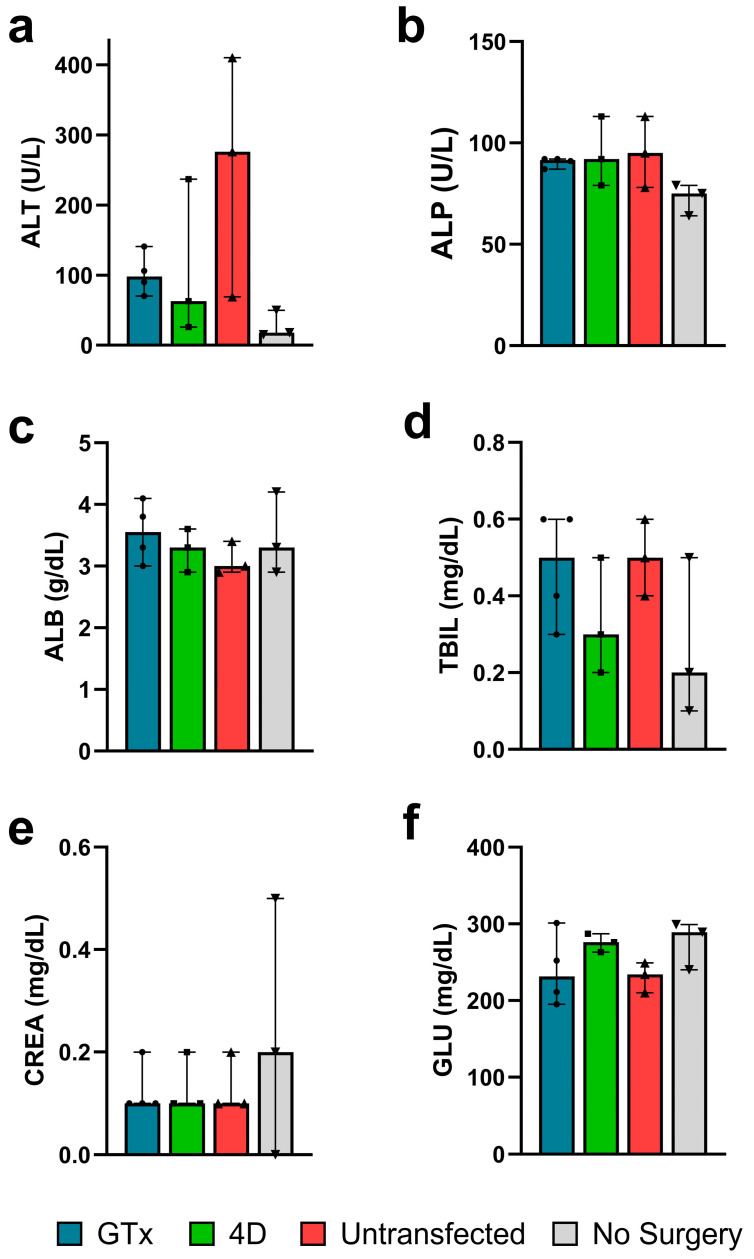
Analyses of liver biochemical markers in *Fah^-/-^* graft recipients. Blood serum concentrations of ALT (**a**), ALP (**b**), ALB (**c**), TBIL (**d**), CREA (**e**), and GLU (**f**) in recipient mice (*n* = 3–4) and no surgery controls (*n* = 3). Bar graphs display the median and biological replicates, and error bars display the range. All sample groups except for CREA levels were observed to take a normal distribution and were analyzed using the Brown–Forsythe and Welch one-way ANOVA with Dunnett’s T3 multiple comparisons significance test. The CREA groups followed a lognormal distribution and were analyzed using the Kruskal–Wallis one-way ANOVA with multiple comparisons significance test. Statistical significance was not observed. Abbreviations: ALT, alanine transaminase; ALP, alkaline phosphatase; ALB, albumin; TBIL, total bilirubin; CREA, creatinine; GLU, glucose.

Segments from the left and right medial lobes were serially sectioned, mounted, and either subjected to IHC staining against Fah to confirm engraftment (Supplementary Figure S4) or H&E staining to assess for potential histological abnormalities. Fah-stained slides further validated high levels of engraftment even in small sections of the outermost portions of the liver for all treatment groups (Figure 3c–e). Aside from mild portal inflammation, no histological abnormalities were observed in the H&E-stained slides for any of the treatment groups, indicating the health of engrafted *mTmG* cells, success of treatment, and overall safety of the therapeutic approach (Supplementary Figure S5). The rest of the liver was homogenized for whole-liver DNA extraction and on-target indel analysis. We observed 53.5%, 58.7%, and 1.0% indels for the GTx, 4D, and untransfected groups, respectively (Figure 3b). Blood serum was collected for liver health biomarker analyses including ALB, ALP, ALT, TBIL, CREA, and GLU. No significant differences were observed in mean biomarker concentrations across groups, including between treatment groups and no surgery controls, indicating a lack of any major complications associated with selection via succinylacetone-induced hepatotoxicity (Figure 4). Interestingly, untransfected controls trended towards greater ALT levels, falling outside of the healthy range for wild-type mice, indicating that they may be experiencing hepatic stress as a result the absence of NTBC (Figure 4a). All mice displayed serum glucose levels well above the normal range (Figure 4f), but this is likely due to the fact that NTBC is supplemented with dextrose due to its unpleasant taste, and mice that were subjected to NTBC withdrawal were kept on dextrose supplementation to prevent any confounding effects.

## 4. Discussion

Our study is the first to demonstrate the performance of the ExPERT GTx electroporator in primary hepatocytes and shows proof-of-principle of its use for treating IMLDs in the *Fah^-/-^* mouse model of HT1. Transplanted *Fah^-/-^* recipients were rescued from acute liver failure and became independent of NTBC, starting twenty days after the splenic injection of electroporated *Fah^+/+^* hepatocytes. Further, the graft recipients exhibited an increase in body weight until the end of this study. An inspection of gross liver anatomy and histological analysis confirmed that, at the end of this study, recipients transplanted with gene-edited electroporated hepatocytes showed no signs of major pathology, fibrosis, or liver damage. This finding indicates that electroporation using the GTx system does not adversely affect hepatocyte functionality or their capacity to engraft and repopulate the liver in *Fah^-/-^* recipient mice.

The transplanted *Fah^+/+^* cells have a natural selective advantage over native mutant *Fah^-/-^* hepatocytes in clonally expanding and replacing diseased hepatocytes when NTBC is withdrawn from recipients, preventing fibrotic lesions and the development of HCC tumors caused by the accumulation of toxic metabolites, including succinylacetone, in the liver [26,27]. The absence of histological abnormality in recipients treated with *Fah^+/+^* hepatocytes electroporated with *Hpd*-aiming Cas9 RNP indicates that the GTx electroporation did not adversely impact the health of the hepatocytes in rescuing the HT1 phenotype. In addition, our results indicate that our cell-based gene editing approach has the potential to benefit HT1 patients who receive late or no NTBC treatment as an alternative to OLT [28,29,30]. It is worth noting that the ex vivo gene editing approach using CRISPR-Cas9 RNPs targeting *Hpd* may result in similar side effects associated with NTBC-induced Hpd inhibition. However, the side effects from Hpd deficiency are mild and manageable [31,32,33,34]. In future studies, we will evaluate the long-term persistence of gene-edited hepatocytes and assess the safety of our approach regarding the risks of developing HCC following the transplantation of hepatocytes electroporated with *Hpd*-aiming Cas9 RNPs using the GTx system.

Therapeutic success was further validated through serum biomarker analyses, whereby serum biomarkers for gene-edited graft recipients were within the normal range (Figure 4), and flow cytometry analysis to quantify engraftment. We observed engraftment levels of 97.9%, 96.8%, and 81.6% for the GTx, 4D, and untransfected groups, respectively (Figure 3a). Our indel analysis confirmed high levels of gene editing efficiency in transplanted hepatocytes and that the edited alleles were retained following clonal expansion in vivo. We observed 53.5%, 58.7%, and 1.0% indels in the liver homogenates from the GTx, 4D, and untransfected recipients, respectively (Figure 3b). Given that hepatocytes account for approximately 60% of the liver by genetic content [35], the levels of gene editing efficiency correspond to 89.1%, 97.9%, and 1.7% for hepatocyte-specific gene editing in the GTx, 4D, and untransfected groups, respectively. The 1.7% editing efficiency observed in the untransfected group is due to background noise, typical for the TIDE tool when analyzing Sanger sequencing traces [36].

The donor hepatocytes used in our studies were isolated from healthy Fah-expressing *mTmG* mice. We hypothesized that all Fah-positive cells would engraft similarly, given that Fah expression prevents intracellular succinylacetone-induced toxicity. Interestingly, we observed higher engraftment (Figure 3a) and lower, although not significant, ALT levels for the electroporated Hpd-deficient hepatocytes than the untransfected control hepatocytes. These results indicate that the transplantation of *Hpd* knockout hepatocytes results in a stronger selective advantage for liver repopulation than wild-type *Hpd^+/+^* hepatocytes in *Fah^-/-^* recipients. Observations in the literature can explain our results. First, it is well documented that *Hpd* deficiency causes global hypertyrosinemia [28,37], and we have previously demonstrated that *Fah^-/-^* recipients transplanted with *Hpd^-/-^* hepatocytes have higher serum tyrosine levels than wild-type mice [25]. It is possible that this hypertyrosinemia caused by the transplantation of Hpd-deficient cells further increased the tyrosine catabolized in the native parenchyma, thereby increasing succinylacetone-induced cytotoxicity and improving the selective advantage of transplanted hepatocytes. Such an event should not occur upon the transplantation of Hpd-expressing *Fah^+/+^* hepatocytes, which would result in less selective pressure and lower engraftment compared to *Hpd^-/-^* hepatocytes.

Secondly, we suspect that electroporation could reduce the immunogenicity of allogeneic hepatocytes. It is well documented that treating cells with electrical currents often brings about membrane-bound protein denaturation, endocytosis, and degradation for peptide recycling [38,39]. Of course, recent technological advancements in electroporators and the optimization of their program voltages effectively limit the denaturation of the lipid-bilayer proteins which are required for cellular survival. However, these proteins are observed to denature at greatly variable electrical intensities [39], and it is possible that those which are inessential to survival are still being destroyed upon treatment. We consider the possibility that major histocompatibility complexes are being denatured and degraded due to electrical shock. Such an event would allow for a more favorable evasion of the host’s immune system upon transplantation and would thus lead to greater engraftment. Future studies investigating the effects of electrical treatment on the immunogenicity of hepatocytes following transplantation could provide greater insights into the impacts of electroporation.

Although CRISPR therapy research is now a topic of great interest, its application under good manufacturing practice (GMP) conditions has greatly limited the progress that can be made toward clinical translation, namely due to exorbitant costs and inefficiencies of vector production and downstream purification, limitations that have only grown more challenging in attempts to upscale [40,41]. However, since it is a physical delivery method, electroporation may serve as a model in which clinical scalability is notably cheaper and more feasible than with viral or lipid nanoparticle vectors, ultimately reducing the cost of potential treatment and making ex vivo gene therapy more mainstream. This is, of course, combined with the fact that ex vivo electroporation does not raise concerns associated with added immunogenicity or insertional mutagenesis and subsequent HCC as commonly observed with various in vivo approaches, such as AAVs and lipid nanoparticles [18,19,20,21,42,43,44]. By observing favorable therapeutic outcomes in using the GMP device, the ExPERT GTx electroporator, these results benchmark the entry of clinically approved electroporation devices for liver-based ex vivo gene therapy. Our results indicate that gene editing using clinical-grade electroporation is a promising approach for IMLDs and has future potential for clinical application.

## Data Availability

The original contributions presented in this study are included in the article/Supplementary Materials. Further inquiries can be directed to the corresponding author.

## Supplementary Materials

The following supporting information can be downloaded at https://www.mdpi.com/article/10.3390/cells14100711/s1. Figure S1: Preliminary optimization of GTx electroporation conditions; Figure S2: Survivorship of Transplant Recipients; Figure S3: Gross liver Images; Figure S4: Anti-Fah-Stained Liver Sections; Figure S5: H&E-Stained Liver Sections.

## Abbreviations

The following abbreviations are used in this manuscript:

IMLD	Inherited metabolic liver disease
EP	Electroporation
HTx	Hepatocyte transplantation
OLT	Orthotopic liver transplantation
HT1	Hereditary tyrosinemia type 1
*Fah*	Fumarylacetoacetate hydrolase
*Hpd*	4-hydroxyphenylpyruvate dioxygenase
HCC	Hepatocellular carcinoma
NTBC	2-(2-nitro-4-trifluoromethyl benzoyl)-1,3-cyclohexanedione
