# Supplementary material for: Validation of Clinical-Grade Electroporation Systems for CRISPR-Cas9-Mediated Gene Therapy in Primary Hepatocytes for the Correction of Inherited Metabolic Liver Disease
Source: Cells. 2025 May 14;14(10):711. doi: 10.3390/cells14100711 (PMC12109753; doi:10.3390/cells14100711)
Supplement: Supplementary file 1 [file cells-14-00711-s001.zip › cells-3590719-supplementary.pdf]

Validation of clinical-grade electroporation systems for CRISPR-Cas9 mediated gene therapy in primary hepatocytes for the correction of inherited metabolic liver disease

Justin Gibson[1§], Abishek Dhungana[1§], Menam Pokhrel[1], Benjamin Arthur[1], Pramita Suresh[1], Olumide Adebayo[1], Renee N. Cottle[1*]

[1] Clemson University, Department of Bioengineering, Clemson, SC

* Correspondence: rcottle@clemson.edu; Tel.: 1-864-656-3071

§ Contributed Equally

Supplemental Figures

Supplemental Figure 1. Preliminary optimization of GTx electroporation conditions.

Supplemental Figure 2. Survivorship of Transplant Recipients.

Supplemental Figure 3. Gross liver Images.

Supplemental Figure 4. Anti-*Fah*-Stained Liver Sections.

Supplemental Figure 5. H&E-Stained Liver Sections.

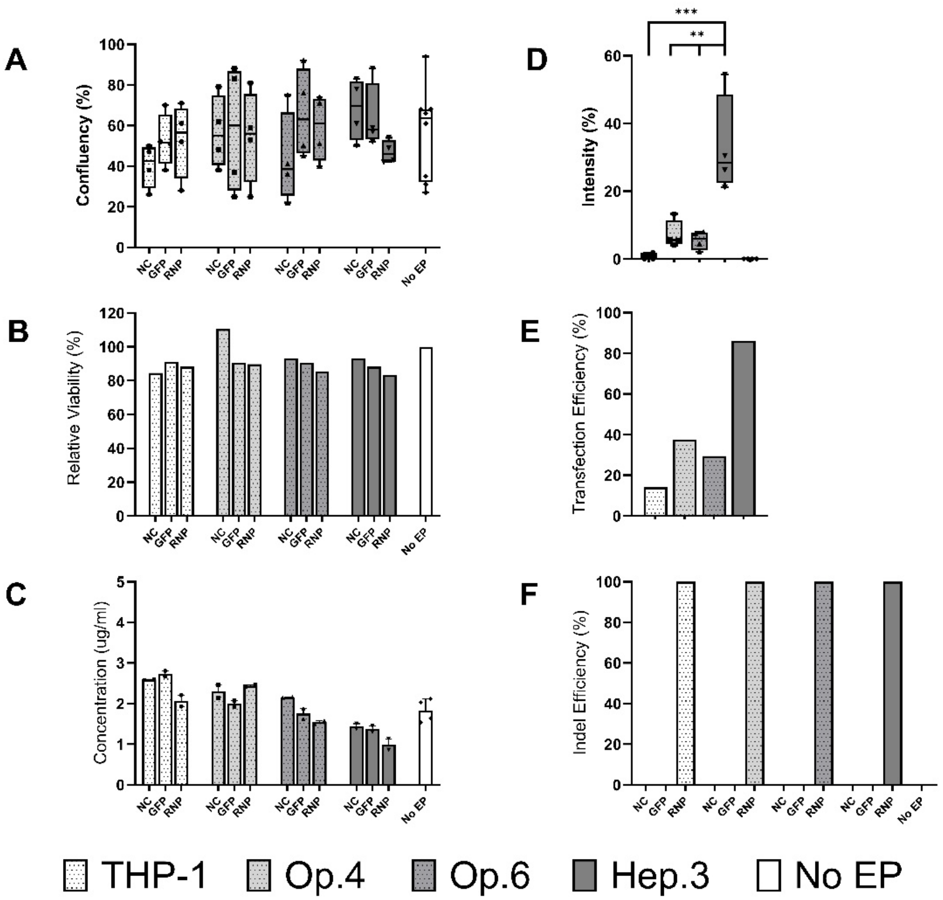

Supplemental Figure 1. Preliminary optimization of GTx electroporation conditions. (A) Percent area of confluency, MTT assay viabilities normalized to no EP control (B), concentration of secreted albumin (C), efficiency of delivery by percent of GFP intensity relative to confluency (D) and efficiency of transfection by percentage GFP positive cells (E), and percentage of gene editing efficiency (F) of hepatocytes plated following electroporation. Plots in A, C, and D represent the median of 2 -4 technical replicates with a 95% confidence interval and the 5-number summary for bar graphs and box and whisker plots, respectively. Statistical significance was denoted as *P<0.05, **P<0.01, and ***P<0.001.

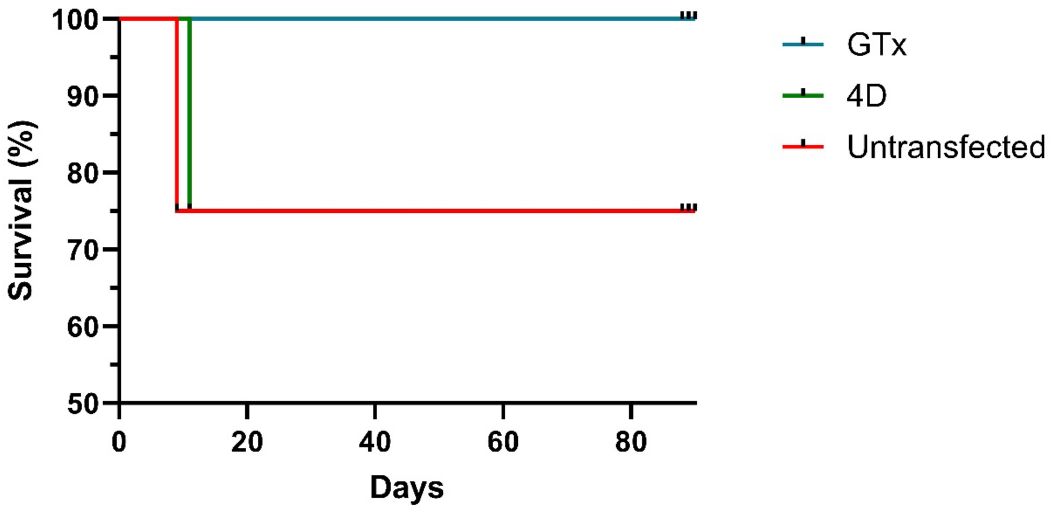

Supplemental Figure 2. Survivorship of Transplant Recipients. Survivorship chart of gene-edited hepatocyte transplantation in *Fah*$^{-/-}$ recipients (n = 4 at day 0).

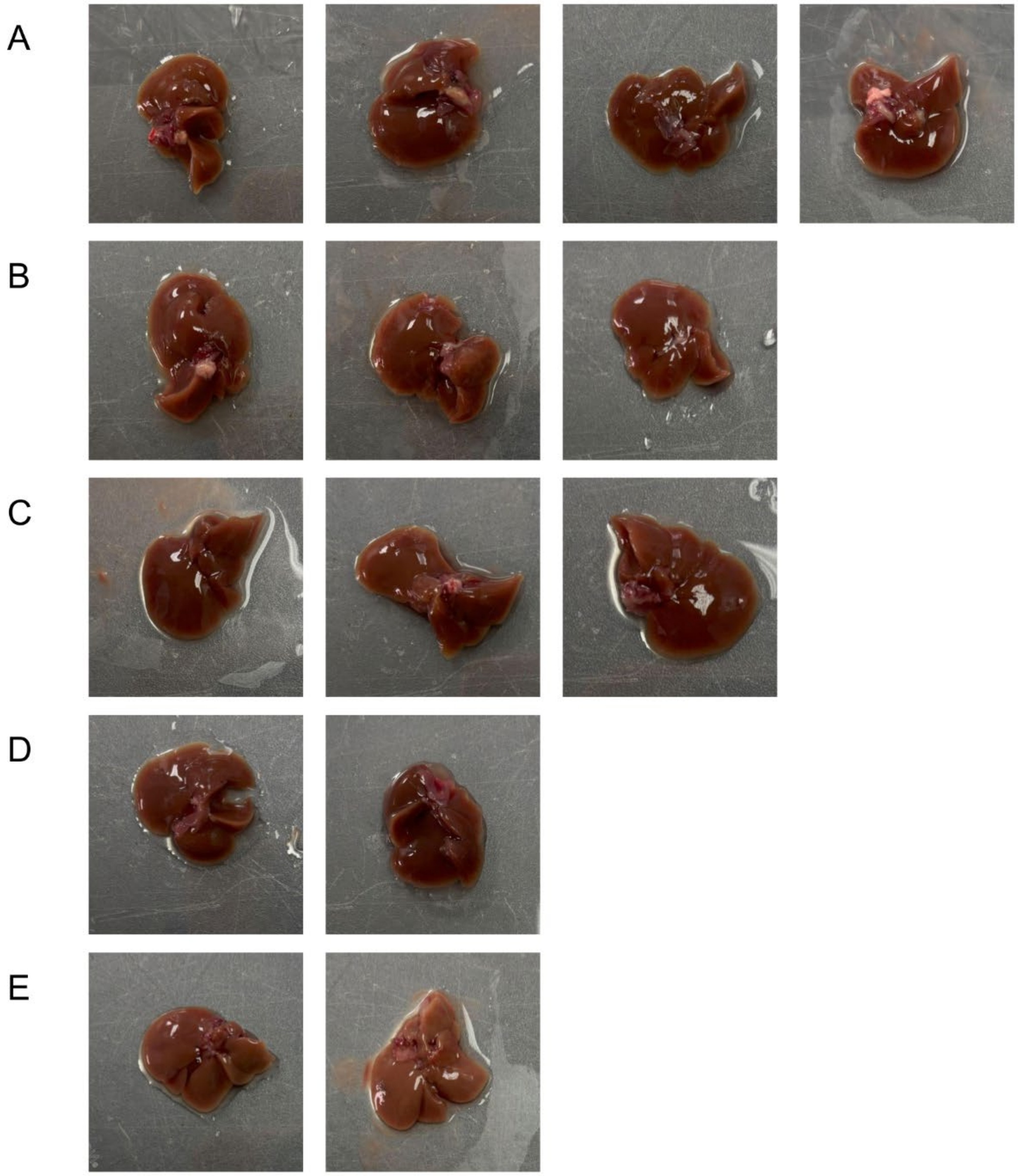

Supplemental Figure 3. Gross liver Images. Gross images of resected livers from GTx (A) (n=4), 4D (B) (n=3), and untransfected (C) (n=3) recipients as well as no-surgery *Fah*$^{-/-}$ controls (D) (n=2) and wild-type no surgery controls (E) (n=2).

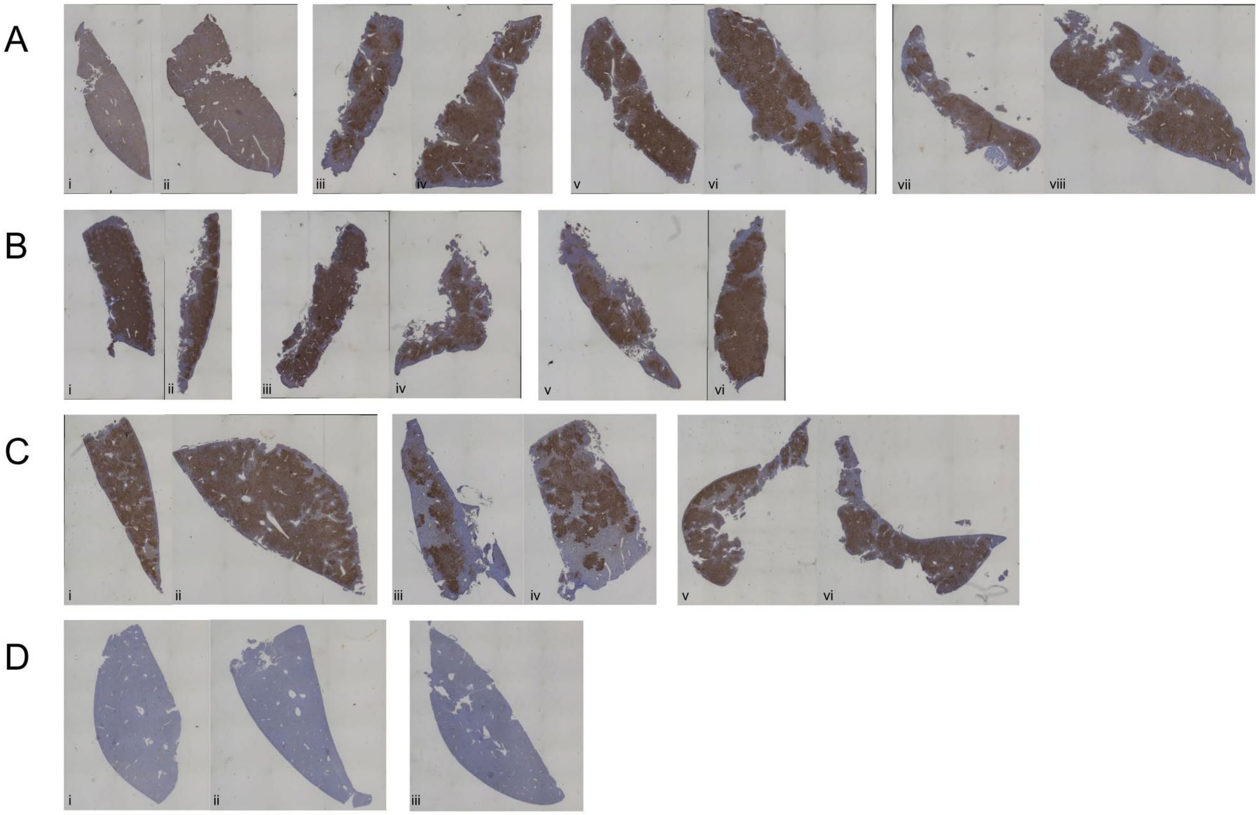

Supplemental Figure 4. Anti-*Fah*-Stained Liver Sections. Anti-*Fah* antibody-stained slides of sectioned livers from GTx (A) (n=4), 4D (B) (n=3), and untransfected (C) (n=3) recipients as well as no-surgery *Fah*$^{-/-}$ controls (D) (n=2).

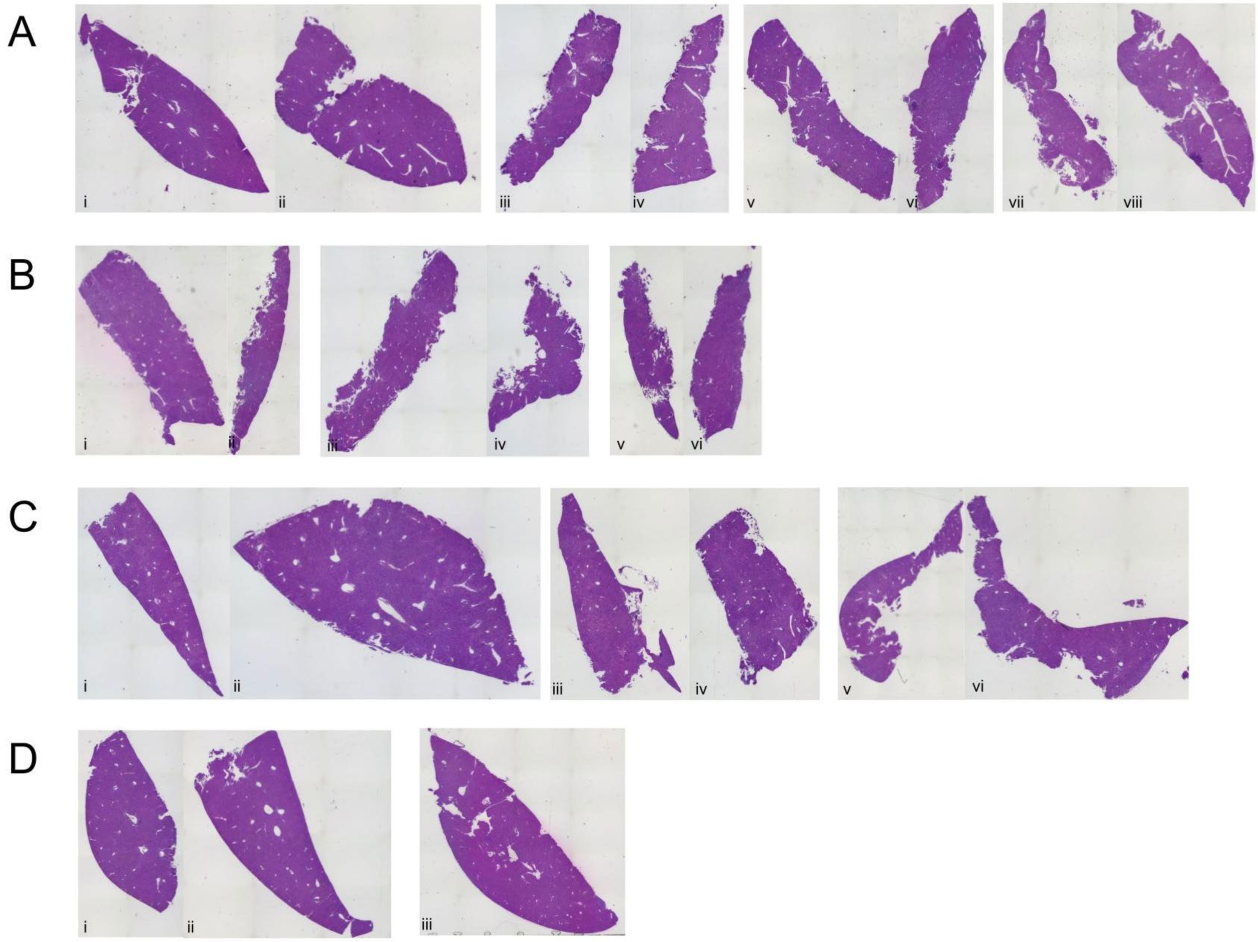

Supplemental Figure 5. H&E-Stained Liver Sections. H&E-stained slides of sectioned livers from GTx (A) (n=4), 4D (B) (n=3), and untransfected (C) (n=3) recipients as well as no-surgery *Fah*$^{-/-}$ controls (D) (n=2).
